# Lithium Toxicity Following Initiation of Semaglutide in a Patient With Bipolar Disorder: A Case Report

**DOI:** 10.7759/cureus.117235

**Published:** 2026-09-30

**Authors:** Annika Dhingra, Charissa M Nichols, Ezekiel Sanchez, Anurag Ayyagari, Yashvi Rateshwar, Nina Narvaez

**Affiliations:** 1 Medicine, Rush University Medical Center, Chicago, USA; 2 Psychiatry, Bergen New Bridge Medical Center, Paramus, USA; 3 Medicine, St. George's University, St. George's, GRD; 4 Academy for Advancement of Science and Technology, Bergen County Academies, Hackensack, USA

**Keywords:** bipolar disorder, case report, drug interaction, glp-1 receptor agonist, lithium toxicity, renal clearance, semaglutide, volume depletion

## Abstract

Glucagon-like peptide-1 (GLP-1) receptor agonists are increasingly prescribed for type 2 diabetes mellitus and obesity, including among patients with severe mental illness. Their effects on gastrointestinal motility, oral intake, and volume status raise concern for interactions with drugs with narrow therapeutic indices that are cleared almost entirely by the kidney, such as lithium. We describe a 69-year-old woman with bipolar disorder, stable for several years on lithium, who presented with acute confusion, dysarthria, and expressive aphasia. A stroke protocol was activated, and noncontrast head CT, CT angiography of the head and neck, and MRI of the brain showed no acute infarct or hemorrhage. Semaglutide had been initiated approximately one month earlier for weight management, and she had experienced substantial weight loss with persistent nausea, intermittent vomiting, and reduced oral intake. Serum lithium was elevated above the reference range, accompanied by a reduced estimated glomerular filtration rate and biochemical findings suggestive of volume depletion. Lithium was held, and intravenous fluids were administered; renal function improved and the neurologic deficits resolved. Lithium was subsequently resumed at a reduced dose without recurrence. In this patient, initiation of semaglutide was temporally associated with lithium toxicity in the setting of gastrointestinal intolerance, reduced oral intake, and suspected volume depletion. Although a single case cannot establish causality, this observation supports closer lithium monitoring when initiating GLP-1 receptor agonists and during dose escalation.

## Introduction

Glucagon-like peptide-1 (GLP-1) receptor agonists have become major therapeutic options for type 2 diabetes mellitus and obesity, with additional cardiovascular and renal benefits [1-3]. Their pharmacologic effects extend beyond glycemic control to include delayed gastric emptying, suppressed appetite, and changes in fluid and sodium handling [4-6].

Patients with bipolar disorder have disproportionately high rates of obesity and type 2 diabetes mellitus, related in part to psychotropic exposure and lifestyle factors [7,8]. GLP-1 receptor agonist use is therefore rising in this population, although available claims data derive from privately insured US adults and do not report lithium co-prescription specifically. In a claims analysis of more than 27 million privately insured US adults, GLP-1 receptor agonist fills among individuals with mood disorders rose from 1.5% in 2018 to 7.2% in 2023, exceeding metformin by the end of that period [9]. Notably, this growth was driven almost entirely by primary care clinicians and nurse practitioners rather than by psychiatrists, whose share of GLP-1 receptor agonist prescribing remained near 6% [9,10]. The clinician initiating the GLP-1 receptor agonist is therefore often not the clinician managing lithium, which creates a structural gap in monitoring.

Lithium remains a cornerstone treatment for bipolar disorder [11]. Still, lithium is eliminated almost entirely by the kidney, is reabsorbed in the proximal tubule in parallel with sodium, and has a narrow therapeutic index [12-14]. Modest reductions in effective circulating volume or glomerular filtration can therefore produce clinically significant increases in serum lithium concentration [13,15]. Because GLP-1 receptor agonists commonly cause nausea, vomiting, and reduced oral intake, and because rapid weight loss may alter volume status, a plausible mechanism for GLP-1-associated lithium accumulation exists [16,17]. No dedicated pharmacokinetic study of lithium with any GLP-1 receptor agonist has been published. Moreover, direct receptor-mediated natriuresis would, if anything, be expected to increase lithium clearance. The proposed mechanism is therefore indirect and uncertain. Reports of this interaction have begun to appear but remain confined to a handful of letters, single cases, and one small series [18-22].

We describe a suspected adverse drug event: symptomatic lithium toxicity in a patient on stable long-term lithium therapy, occurring one month after semaglutide initiation and presenting as a suspected stroke, with laboratory findings suggestive of volume depletion that improved with intravenous fluids.

## Case presentation

A 69-year-old woman with a history of bipolar disorder, alcohol use disorder in remission, and hypothyroidism was brought to the emergency department by emergency medical services after family observed acute confusion and slurred speech.

On arrival, she was alert and oriented to person, place, and time. Vital signs were temperature 36.3°C, heart rate 74 beats per minute, blood pressure 118/61 mmHg, respiratory rate 18 breaths per minute, and oxygen saturation 98% on room air. Her weight was 75.3 kg. Neurologic examination demonstrated dysarthria and expressive aphasia with intact motor function and preserved ability to follow commands. The remainder of the examination was unremarkable. Tremor, ataxia, clonus, hyperreflexia, and myoclonus were not documented, and no formal cognitive testing was performed.

A stroke protocol was activated. Initial laboratory studies showed leukocytosis with neutrophilic predominance, a mildly reduced estimated glomerular filtration rate, low serum bicarbonate, and microcytic anemia with normal iron studies. Urinalysis showed proteinuria with pyuria and hematuria, without nitrites or leukocyte esterase. A urine toxicology screen was negative for amphetamines, barbiturates, benzodiazepines, cannabinoids, cocaine, opiates, and phencyclidine, and the serum alcohol level was below the limit of detection. Thyroid function testing showed a suppressed thyroid-stimulating hormone with an elevated free thyroxine, consistent with over-replacement rather than the documented history of hypothyroidism.

Noncontrast CT of the head demonstrated no intracranial hemorrhage, mass effect, or midline shift within the limits of the study; evaluation of the posterior fossa was limited by streak artifact. Periventricular chronic small-vessel ischemic changes and mild volume loss were present, with an Alberta Stroke Program Early CT Score (ASPECTS) of 10. CT angiography of the head and neck, performed with intravenous iodinated contrast, showed atheromatous calcification of the cavernous and supraclinoid segments of both internal carotid arteries, with no large-vessel occlusion, critical stenosis, dissection, or aneurysm; the left vertebral artery was dominant. MRI of the brain without contrast showed no restricted diffusion to suggest acute infarction and no hemorrhage, mass, or midline shift, with mild volume loss and chronic small-vessel ischemic change. Transthoracic echocardiography with bubble study showed a left ventricular ejection fraction of 55-60% with an impaired relaxation filling pattern, moderate tricuspid regurgitation, an estimated pulmonary artery pressure of 42 mmHg, and no patent foramen ovale.

Given leukocytosis (17.9 × 10³/mm³) and pyuria on urinalysis, bacterial infection was considered a possible contributor to her acute presentation, with a respiratory or urinary source suspected; the patient received empiric intravenous vancomycin (1 g) and piperacillin-tazobactam (3.375 g). She was admitted to the neurology service with a working diagnosis of acute stroke. Psychiatry was consulted for a possible functional neurologic disorder after imaging returned negative.

Collateral history subsequently clarified the medication regimen, which had been stable for several years: lithium immediate-release 300 mg twice daily, bupropion XL 450 mg daily, gabapentin 300 mg three times daily, clonazepam 0.5 mg twice daily as needed for anxiety, and zolpidem 10 mg nightly as needed for insomnia. Approximately one month before presentation, semaglutide 0.25 mg subcutaneously weekly had been initiated for weight management. Since starting semaglutide, she had lost approximately 13.6 kg (30 lb) and had persistent nausea with intermittent vomiting and reduced oral intake.

A serum lithium concentration measured early on hospital day 2, about eight hours after the initial emergency department laboratory studies, returned at 1.7 mmol/L (reference range 0.5-1.5 mmol/L; critical value >2.0 mmol/L). The timing of the last lithium dose was not recorded, so the sample cannot be confirmed as a predose trough, and the concentration is reported descriptively rather than used to grade severity; toxicity was characterized clinically, based on acute confusion reported by family, dysarthria, and expressive aphasia in a patient on chronic therapy. Lithium was held, and intravenous crystalloid was administered.

Hemoglobin fell from 11.4 g/dL on the initial emergency department sample to 10.0 g/dL on the hospital day 2 sample, and hematocrit from 38.0% to 33.9%, without bleeding. Between hospital days 2 and 3, serum creatinine fell from 1.16 to 0.79 mg/dL, estimated glomerular filtration rate rose from 51 to 81 mL/min/1.73 m², and serum lithium decreased from 1.7 to 1.2 mmol/L, then rose to 1.3 mmol/L on hospital day 4. Leukocytosis downtrended from 17.9 to 10.5 × 10³/mm³. Procalcitonin was <0.05 ng/mL, and MRSA PCR was negative; a respiratory pathogen panel on hospital day 2 and a sputum pneumonia panel on hospital day 3 detected human rhinovirus/enterovirus, with no bacterial targets. The leukocytosis was therefore attributed to a reactive process rather than bacterial infection, and antibiotics were discontinued.

Clinically, the dysarthria and expressive aphasia resolved, and speech returned to baseline fluency. At discharge, the patient was at her cognitive and functional baseline with normal speech, ambulation, and oral intake.

Outcome and follow-up

The patient was discharged with close outpatient psychiatric follow-up for lithium dose adjustment, serial monitoring of serum lithium and renal function during continued GLP-1 receptor agonist therapy, emphasis on adequate hydration and nutritional intake (particularly during dose escalation), and outpatient reassessment of thyroid function given the biochemical pattern of levothyroxine over-replacement. Lithium was subsequently resumed at a reduced dose of 150 mg twice daily, without recurrence of toxicity.

Laboratory findings

All laboratory values obtained during the admission are presented in Tables 1-5 with the corresponding electronic health record (EHR) reference ranges. Values outside the reference range are flagged (H = high, L = low, A = abnormal). Table 6 summarizes imaging findings.

**Table 1:** Serum lithium and routine chemistry Serial chemistry across admission. eGFR was reduced at presentation and on day 2, then improved after intravenous fluid administration, with a parallel fall in serum creatinine. Serum bicarbonate was low at presentation; the accompanying hyperchloremia developed on days 2 and 3 and had not resolved by the final measurement. eGFR: estimated glomerular filtration rate, BUN: blood urea nitrogen, AST: aspartate aminotransferase, ALT: alanine aminotransferase, H: above reference range, L: below reference range, - indicates the test was not performed on that hospital day.​​​​

Test	Unit	EHR reference range	Day 1	Day 2	Day 3	Day 4
Lithium	mmol/L	0.5-1.5; critical >2.0	-	1.7 (H)	1.2	1.3
Sodium	meq/L	135-145	137	135	137	-
Potassium	meq/L	3.5-5.0	4.8	4.1	4.0	-
Chloride	meq/L	98-107	107	109 (H)	108 (H)	-
CO₂	meq/L	21-31	20 (L)	18 (L)	23	-
Glucose	mg/dL	70-110	133 (H)	101	101	-
Calcium	mg/dL	8.6-10.3	9.5	8.3 (L)	9.1	-
BUN	mg/dL	7-23	19	21	10	-
Creatinine	mg/dL	0.6-1.3	1.06	1.16	0.79	-
eGFR (creatinine)	mL/min/1.73 m²	>60	57 (L)	51 (L)	81	-
Total bilirubin	mg/dL	0.3-1.1	1.0	0.7	0.6	-
Total protein	g/dL	6.4-8.4	6.5	5.9 (L)	6.1 (L)	-
Albumin	g/dL	3.5-5.7	4.3	3.9	4.0	-
Alkaline phosphatase	unit/L	34-104	118 (H)	101	109 (H)	-
AST	unit/L	13-39	14	14	14	-
ALT	unit/L	7-52	13	14	14	-
Phosphorus	mg/dL	2.5-5.0	-	4.0	2.9	-
Magnesium	mg/dL	1.7-2.5	-	2.4	2.2	-
Transferrin	mg/dL	205-362	-	229	-	-
C-reactive protein	mg/L	<9.9	-	<5.0	-	-
Lactic acid	mmol/L	0.5-2.2	1.5	-	-	-
Hemoglobin A1c	%	4-6	-	5.6	-	-

**Table 2:** Complete blood count Leukocytosis at presentation resolved without a bacterial source identified on molecular testing, and hemoglobin and hematocrit declined between the initial emergency department sample and hospital day 2, which may reflect dilution or positional and sampling variation.​​​​ The microcytic indices were stable and accompanied by normal iron studies. WBC: white blood cell count, RBC: red blood cell count, MCV: mean corpuscular volume, MCH: mean corpuscular hemoglobin, MCHC: mean corpuscular hemoglobin concentration, RDW: red cell distribution width, MPV: mean platelet volume, - indicates the test was not performed on that hospital day

Test	Unit	EHR reference range	Day 1	Day 2	Day 3
WBC	×10³/mm³	4.5-11.0	17.9 (H)	14.5 (H)	10.5
RBC	×10⁶/mm³	4.00-5.33	5.74 (H)	5.10	5.27
Hemoglobin	g/dL	12.0-16.0	11.4 (L)	10.0 (L)	10.6 (L)
Hematocrit	%	36.0-46.0	38.0	33.9 (L)	34.5 (L)
MCV	fL	80.0-100.0	66.2 (L)	66.5 (L)	65.5 (L)
MCH	pg	26.0-32.0	19.9 (L)	19.6 (L)	20.1 (L)
MCHC	g/dL	31.0-37.0	30.0 (L)	29.5 (L)	30.7 (L)
RDW	%	0.5-16.5	18.6 (H)	18.1 (H)	17.9 (H)
Platelets	K/mm³	140-440	317	300	305
MPV	fL	7.4-10.4	10.9 (H)	10.3	10.9 (H)

**Table 3:** Leukocyte differential Neutrophil-predominant leukocytosis with relative lymphopenia, a nonspecific stress or reactive pattern. - indicates the test was not performed on that hospital day

Test	Unit	EHR reference range	Day 1	Day 2	Day 3
Neutrophils, automated	%	36-75	-	80.0 (H)	-
Lymphocytes, automated	%	24-44	-	7.8 (L)	-
Monocytes, automated	%	4-10	-	8.7	-
Eosinophils, automated	%	0-5	-	2.8	-
Basophils, automated	%	0-2	-	0.3	-
Immature granulocytes	%	0.0-0.6	-	0.4	-
Nucleated RBC	%	0.0	0.0	0.0	0.0
Segmented neutrophils, manual	%	36-75	91 (H)	-	87 (H)
Bands, manual	%	0-10	0	-	2
Lymphocytes, manual	%	24-44	2 (L)	-	3 (L)
Monocytes, manual	%	4-10	5	-	4
Eosinophils, manual	%	0-5	1	-	4
Basophils, manual	%	0-2	1	-	0
Absolute neutrophils, automated	×10³/mm³	1.30-7.80	-	11.62 (H)	-
Absolute neutrophils, manual	×10³/mm³	1.30-7.80	16.29 (H)	-	9.34 (H)
Absolute lymphocytes, automated	×10³/mm³	0.9-4.6	-	1.13	-
Absolute lymphocytes, manual	×10³/mm³	0.9-4.6	0.36 (L)	-	0.32 (L)
Absolute monocytes, automated	×10³/mm³	0.2-1.0	-	1.27 (H)	-
Absolute monocytes, manual	×10³/mm³	0.2-1.0	0.90	-	0.42
Absolute eosinophils, automated	×10³/mm³	0-1	-	0.41	-
Absolute eosinophils, manual	×10³/mm³	0-1	0.18	-	0.42
Absolute basophils, automated	×10³/mm³	0-0.2	-	0.04	-
Absolute basophils, manual	×10³/mm³	0-0.2	0.18	-	0.00

**Table 4:** Peripheral blood smear morphology Peripheral smear findings on hospital days 1 and 3. Platelets were adequate on both slides. Red cell changes were stable and consistent with the microcytic indices in Table 2; no findings suggested a microangiopathic process requiring further evaluation. - indicates the finding was not reported or no smear was reviewed on that hospital day

Morphologic finding	Day 1	Day 2	Day 3
Platelet estimate	Adequate	-	Adequate
Microcytes	Slight	-	Moderate
Hypochromia	-	-	Moderate
Polychromasia	-	-	Slight
Burr cells	Few	-	Few
Ovalocytes	Few	-	Few
Elliptocytes	Few	-	-
Schistocytes	-	-	Few
Target cells	-	-	Few
Teardrop cells	Few	-	-

**Table 5:** Additional serum studies, urinalysis, and microbiology Thyroid function testing showed a suppressed TSH with elevated free T4, consistent with levothyroxine over-replacement. Toxicology was negative, and molecular testing detected only a respiratory virus, with procalcitonin below the limit of detection. TIBC: total iron-binding capacity, TSH: thyroid-stimulating hormone, PT: prothrombin time, INR: international normalized ratio, PTT: partial thromboplastin time, A: abnormal, - indicates not applicable or not reported​​

Category	Test	Hospital day	Result	Unit	EHR reference range
Iron studies	Iron	Day 2	100	mcg/dL	50-212
Iron studies	TIBC	Day 2	341	mcg/dL	250-400
Iron studies	Iron saturation	Day 2	29	%	15-50
Iron studies	Ferritin	Day 2	142.0	ng/mL	14.0-233.0
Nutrition	Folate	Day 2	>22.3	ng/mL	≥4.1
Nutrition	Vitamin B12	Day 1	711	pg/mL	211-911
Thyroid	Free T3	Day 1	2.76	pg/mL	Not reported
Thyroid	Free T4	Day 1	1.84 (H)	ng/dL	0.61-1.12
Thyroid	TSH	Day 1	0.123 (L)	mcIU/mL	0.450-5.330
Cardiac	High-sensitivity troponin I	Day 1	4	pg/mL	3-17; critical >100
Lipids	Total cholesterol	Day 1	129	mg/dL	≤199
Lipids	Triglycerides	Day 1	96	mg/dL	≤149
Lipids	VLDL	Day 1	19	mg/dL	Not reported
Lipids	HDL	Day 1	53	mg/dL	Not reported
Lipids	Direct LDL	Day 1	57	mg/dL	≤129
Coagulation	PT	Day 1	14.8	sec	Not reported
Coagulation	INR	Day 1	1.1	-	Not reported
Coagulation	PTT	Day 1	22.0	sec	Not reported
Toxicology	Serum alcohol	Day 1	<10	mg/dL	-
Toxicology	Urine drug screen (amphetamine, barbiturate, benzodiazepine, THC, cocaine, opiate, PCP)	Day 2	All negative	-	-
Urinalysis	Color/appearance	Day 2	Yellow/clear	-	-
Urinalysis	pH	Day 2	6.0	-	-
Urinalysis	Specific gravity	Day 2	1.020	-	-
Urinalysis	Glucose, bilirubin, ketones, blood, urobilinogen, nitrite, leukocyte esterase	Day 2	All negative	-	-
Urinalysis	Dipstick protein	Day 2	30 (A)	mg/dL	-
Urinalysis	Microscopy: WBC	Day 2	11-20 (A)	/hpf	-
Urinalysis	Microscopy: RBC	Day 2	21-50 (A)	/hpf	-
Microbiology	Procalcitonin	Day 2	<0.05	ng/mL	-
Microbiology	MRSA PCR	Day 2	Negative	-	-
Microbiology	Respiratory pathogen panel	Day 2	Human rhinovirus/enterovirus detected	-	-
Microbiology	Sputum pneumonia panel	Day 3	Human rhinovirus/enterovirus detected; resistance genes not reported	-	-

**Table 6:** Imaging and other diagnostic studies ASPECTS: Alberta Stroke Program Early CT Score, LVEF: left ventricular ejection fraction

Hospital day	Study	Documented findings
Day 1	Noncontrast CT head	Posterior fossa evaluation limited by streak artifact. No intracranial hemorrhage, mass effect, or midline shift to the extent visualized. Periventricular chronic small-vessel ischemic change and mild volume loss. ASPECTS 10.
Day 1	CT angiography, head and neck	Atheromatous calcification of the cavernous and supraclinoid segments of both internal carotid arteries. No large-vessel occlusion, critical stenosis, dissection, or aneurysm. Left vertebral artery dominant. Incidental small emphysematous bullae at the right lung apex and degenerative spinal changes.
Day 2	Transthoracic echocardiogram with bubble study	LVEF 55-60%; borderline left ventricular hypertrophy; impaired relaxation pattern of diastolic filling; sigmoid septum; mild mitral regurgitation; moderate tricuspid regurgitation; estimated pulmonary artery pressure 42 mmHg suggesting moderate pulmonary hypertension; mild aortic sclerosis without stenosis; trace pulmonic regurgitation; no patent foramen ovale.
Day 3	MRI brain without contrast	No restricted diffusion to suggest acute infarct. No hemorrhage, mass, or midline shift. Mild volume loss and chronic small-vessel ischemic changes.

Figure 1 shows representative images from the stroke workup. Figure 2 shows diffusion-weighted imaging and the corresponding apparent diffusion coefficient map. Figure 3 shows T2 FLAIR imaging. Figure 4 shows the lithium time course over the admission.

**Figure 1:**
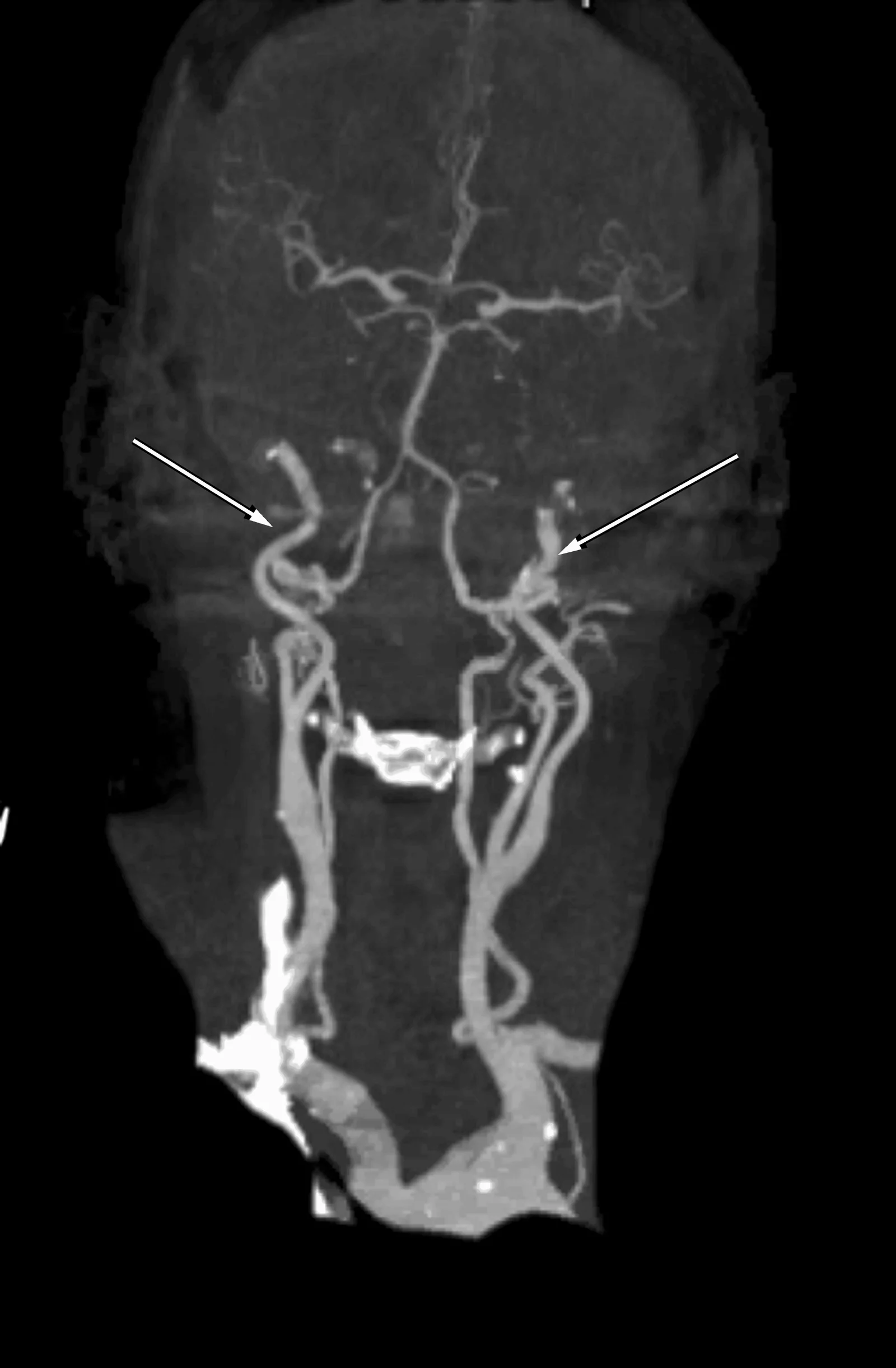
CT angiography of the head and neck Coronal maximum-intensity projection, hospital day 1. Arrows indicate atheromatous calcification of the cavernous and supraclinoid segments of both internal carotid arteries. The cervical and intracranial vessels are otherwise patent without large-vessel occlusion. CT: computed tomography

**Figure 2:**
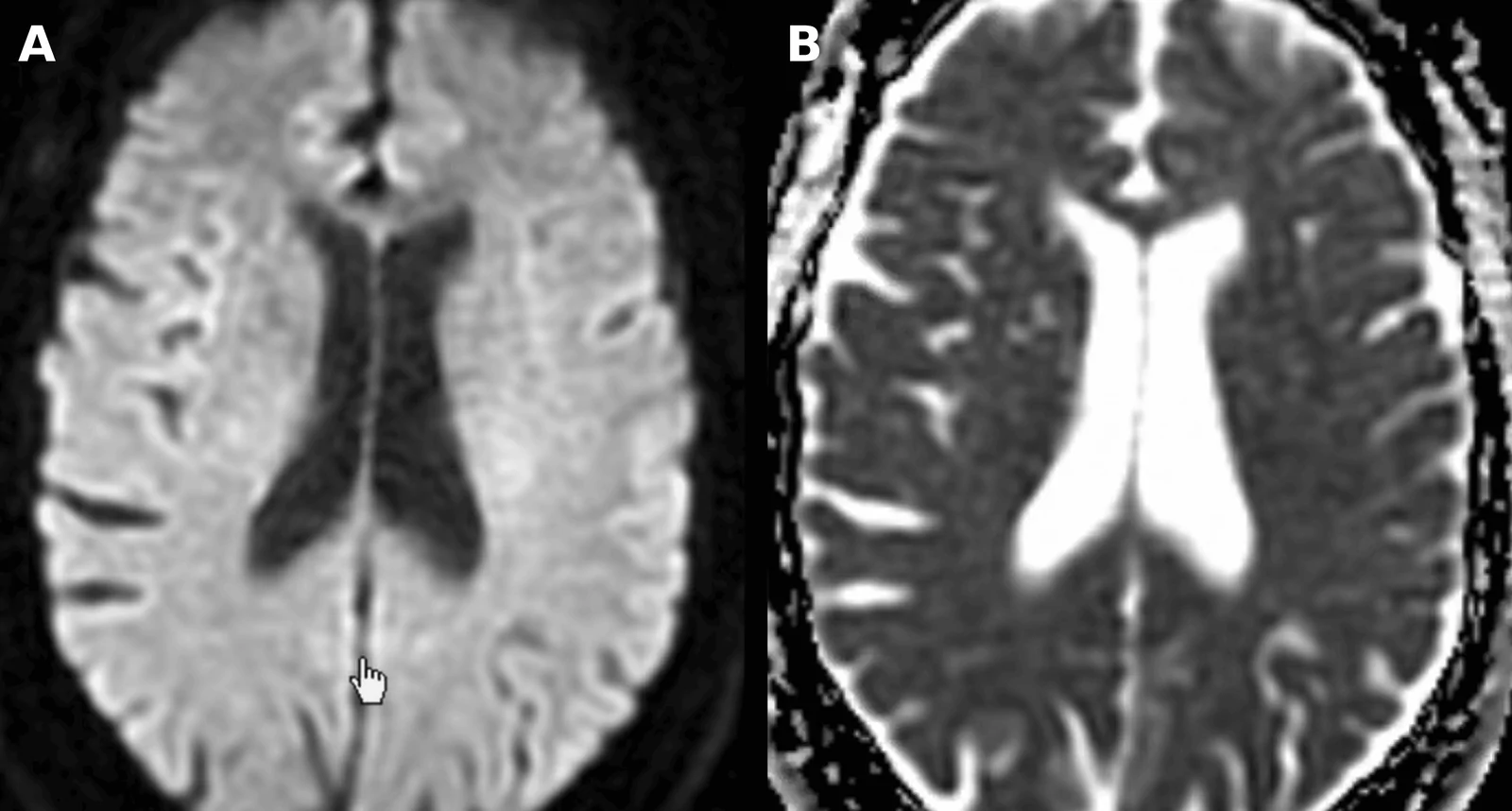
MRI brain diffusion-weighted imaging and corresponding apparent diffusion coefficient map Axial cross-section at the level of the lateral ventricles, hospital day 3. (A) Diffusion-weighted image. (B) Apparent diffusion coefficient map. No focus of restricted diffusion is present, and there is no corresponding reduction in diffusivity; no region of interest is annotated, as the study demonstrates no acute infarction. MRI: magnetic resonance imaging

**Figure 3:**
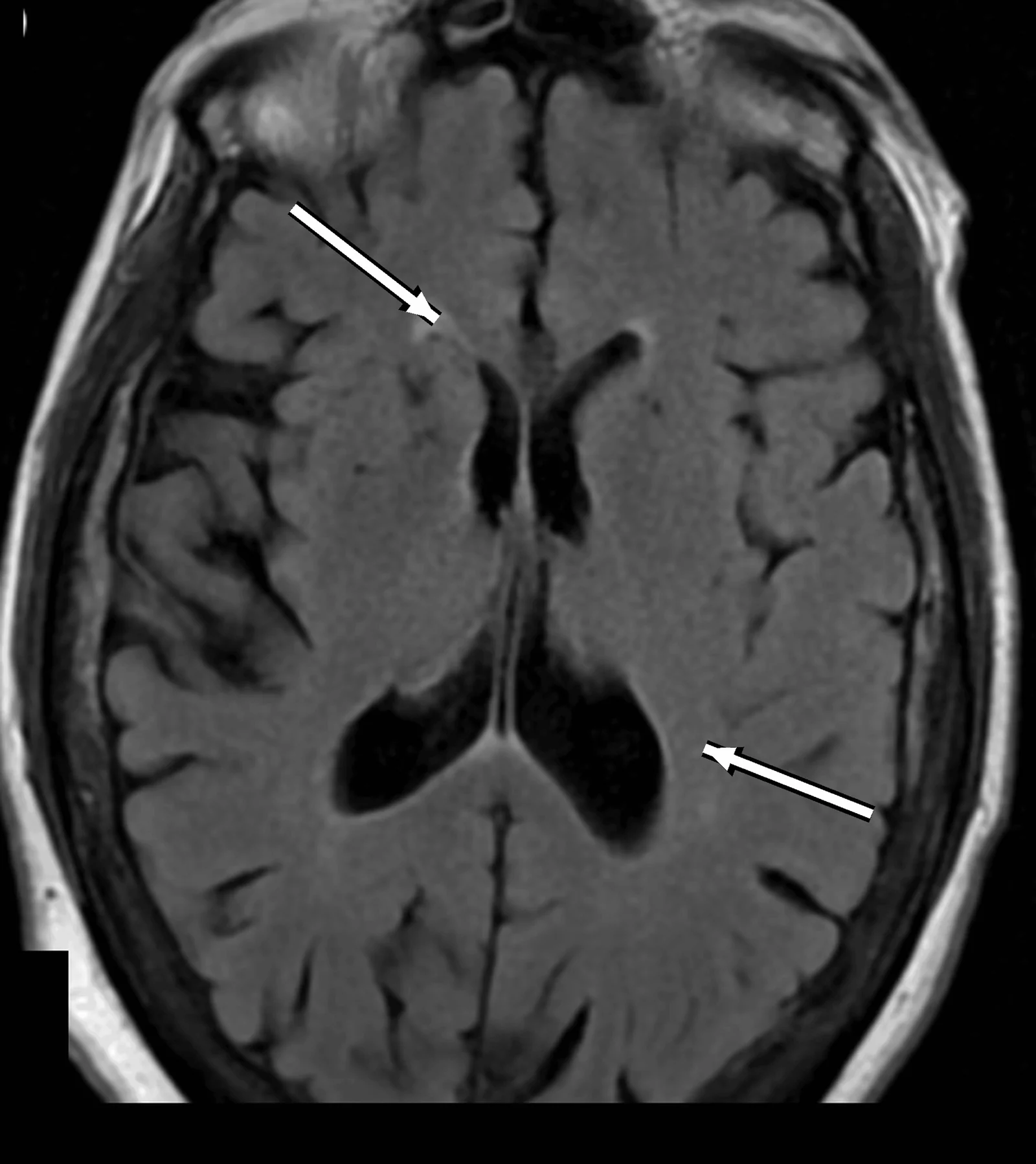
MRI brain T2 fluid-attenuated inversion recovery Axial cross-section at the level of the lateral ventricles, hospital day 3. Arrows indicate periventricular hyperintensity consistent with chronic small-vessel ischemic change. No acute abnormality is seen. FLAIR: fluid-attenuated inversion recovery, MRI: magnetic resonance imaging

**Figure 4:**
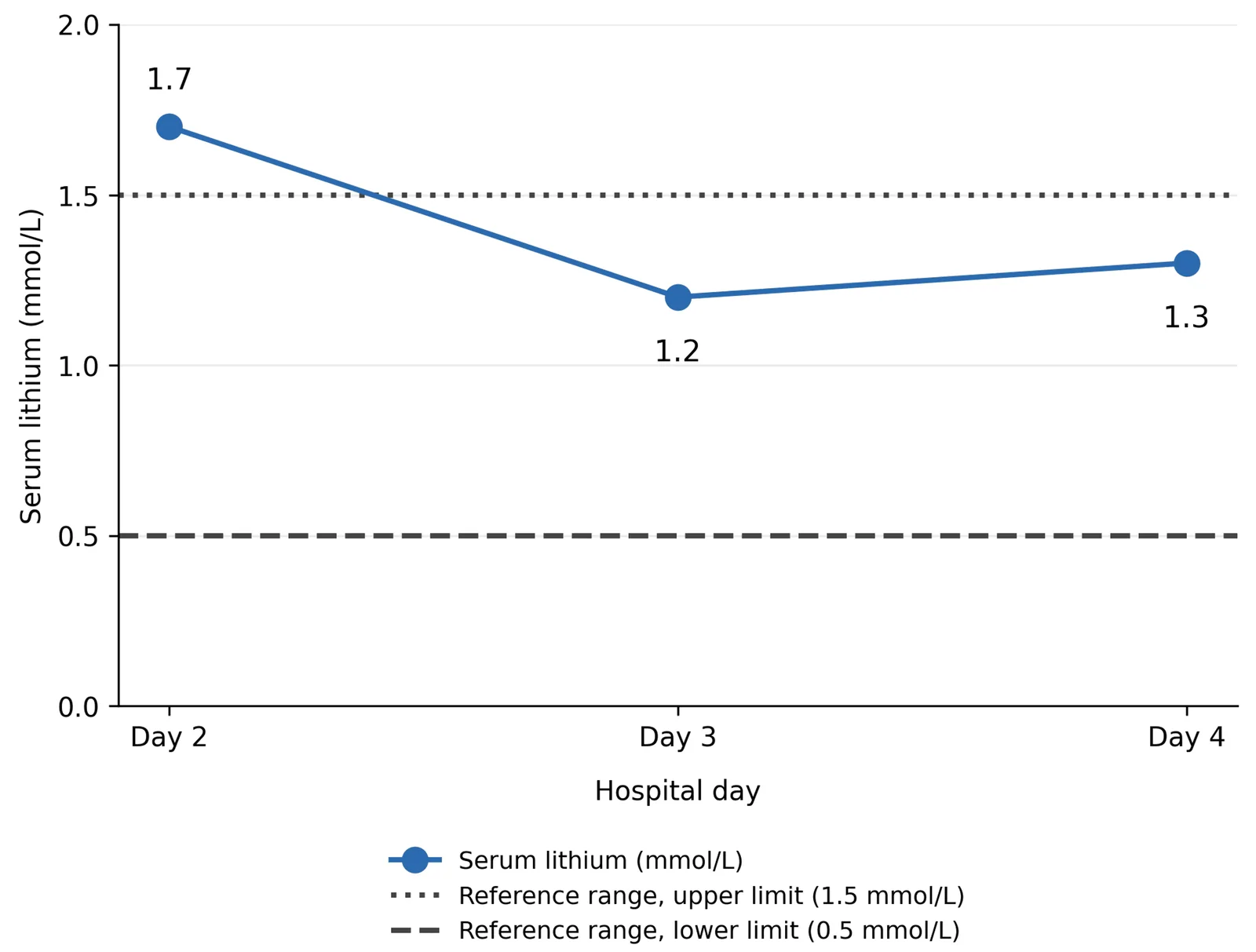
Serum lithium concentration during hospitalization Serum lithium was 1.7 mmol/L at diagnosis, fell to 1.2 mmol/L after lithium was held and intravenous fluids were administered, and was 1.3 mmol/L the following day. Dotted and dashed lines indicate the upper and lower limits of the laboratory reference range (0.5-1.5 mmol/L), respectively. Neurologic symptoms resolved over this interval despite the concentration remaining above the usual maintenance target, consistent with the recognized dissociation between serum lithium concentration and central nervous system tissue burden.

## Discussion

This case describes symptomatic lithium toxicity emerging one month after initiation of semaglutide in a patient whose lithium dose had been unchanged for years. The presentation was mistaken for acute stroke, and the diagnosis was made only after collateral history disclosed the new GLP-1 receptor agonist and the associated gastrointestinal intolerance and weight loss.

Mechanism of the interaction

Lithium is not protein-bound, is not metabolized, and is eliminated almost exclusively by glomerular filtration, with roughly 60-80% of the filtered load reabsorbed in the proximal tubule by the same sodium transport pathways that handle sodium [12,13,23]. Renal lithium clearance is therefore tightly coupled to effective circulating volume and to proximal sodium avidity and is used experimentally as a marker of proximal tubular sodium handling. In states of volume contraction, proximal sodium reabsorption increases and lithium reabsorption increases in parallel, so serum lithium rises for reasons that are largely independent of dose [13,14]. This same mechanism underlies the well-described interactions between lithium and thiazide diuretics, angiotensin-converting enzyme inhibitors, angiotensin receptor blockers, and nonsteroidal anti-inflammatory drugs [24].

GLP-1 receptor agonists do not interact with lithium via cytochrome P450 metabolism or plasma protein binding. Dedicated pharmacokinetic studies of semaglutide with co-administered drugs have shown no clinically meaningful effects on systemic exposure for metformin, warfarin, atorvastatin, or digoxin, and a systematic review of GLP-1 receptor agonist drug-drug interactions with oral medications found few clinically significant signals [25,26]. Lithium has never been formally studied in this way. The interaction described here is therefore best understood as physiologic rather than pharmacokinetic in the conventional sense.

An important nuance is that the direct renal effect of GLP-1 receptor agonism would be expected to oppose the mechanism underlying the observed toxicity. GLP-1 receptor stimulation inhibits the sodium-hydrogen exchanger isoform 3 (NHE3) in the proximal tubule, reducing proximal sodium reabsorption and producing natriuresis; in human studies, prolonged GLP-1 receptor agonist therapy increases urinary lithium excretion, and GLP-1 receptor antagonism conversely reduces lithium clearance and increases NHE3 phosphorylation [27-29]. GLP-1 receptor agonism is also associated with pro-atrial natriuretic peptide release [30] and has demonstrated renoprotective effects on hard renal outcomes in cardiovascular outcome trials [31]. A purely receptor-mediated renal effect would therefore be expected to lower, not raise, serum lithium. Consistent with this, a population-level analysis using the TriNetX network reported fewer renal events in patients receiving GLP-1 receptor agonists alongside lithium than in patients on lithium alone. However, that analysis was not peer-reviewed at the time of writing and did not evaluate pharmacokinetic interactions [32].

This makes the indirect gastrointestinal pathway the more plausible explanation for clinically manifest toxicity. Semaglutide reduces appetite and delays gastric emptying, and nausea and vomiting occur in a substantial minority of patients, particularly during dose escalation [16,17]. The approximately 30-pound weight loss observed in this patient is within the range reported in semaglutide trials and does not, by itself, indicate excessive dosing [33]. The combination of reduced oral fluid intake, ongoing gastrointestinal losses, and rapid weight loss can produce a contracted extracellular volume, a fall in glomerular filtration, and increased proximal tubular sodium and lithium reabsorption sufficient to overwhelm any direct natriuretic effect. Framing the interaction this way also predicts who is at risk: not every patient on both drugs, but specifically those with significant gastrointestinal intolerance, rapid weight loss, reduced renal reserve, or concurrent medications that impair renal lithium handling.

The laboratory data in this case are consistent with that sequence. The estimated glomerular filtration rate was 57 mL/min/1.73 m² at presentation and 51 mL/min/1.73 m² on hospital day 2, then rose to 81 mL/min/1.73 m² on hospital day 3 as serum creatinine fell from 1.16 to 0.79 mg/dL and serum lithium fell from 1.7 to 1.2 mmol/L. This parallel recovery of renal function and lithium concentration after holding lithium and giving intravenous fluids is the strongest evidence in this case for a reversible prerenal component. Other findings are less specific. Hemoglobin and hematocrit fell between the emergency department and hospital day 2 samples without any source of bleeding, which is compatible with dilution of hemoconcentrated values but may also reflect positional or sampling variation; dilutional change alone does not establish antecedent volume depletion. Serum bicarbonate was low at presentation with a normal anion gap, and the hyperchloremia that developed on hospital days 2 and 3 followed crystalloid administration and may reflect chloride-rich fluids rather than the underlying illness. Iodinated contrast administered for CT angiography on hospital day 1 may also have contributed to the small rise in creatinine between days 1 and 2. Taken together, these findings support transient prerenal impairment, rather than a change in lithium dosing or adherence, as the most plausible contributor to the elevated concentration. However, they do not exclude other contributors.

A secondary, less certain contributor is delayed gastric emptying. Immediate-release lithium carbonate is normally absorbed rapidly with a time to peak concentration of approximately 0.5-3 hours. Slowed gastric emptying could shift and blunt the absorption peak, complicating trough monitoring and, in principle, altering both the timing of peak concentration and total exposure [16]. Whether extended-release formulations behave differently under GLP-1 receptor agonist therapy is unknown and is a reasonable target for pharmacokinetic study.

Clinical presentation and differential diagnosis

Neurotoxicity is the dominant manifestation of lithium toxicity and can include confusion, dysarthria, tremor, ataxia, myoclonus, hyperreflexia, and, in severe cases, seizures and encephalopathy [13,14]. Focal or focal-appearing deficits such as expressive aphasia are less commonly emphasized but are reported, and they are the reason lithium toxicity is a recognized stroke mimic. In this patient, an initial code stroke evaluation with noncontrast CT, CT angiography, and subsequent MRI was entirely negative for acute infarction or hemorrhage, and a functional neurologic disorder was considered before the lithium concentration was known.

Several features of this case deserve emphasis for the clinician confronted with a similar presentation. First, the serum lithium level was not obtained until the second hospital day, after an extensive and negative stroke workup and after empiric broad-spectrum antibiotics had been administered. In any patient on chronic lithium presenting with acute encephalopathy or dysarthria, a lithium concentration is inexpensive, rapid, and should be part of the first-line panel. Second, the degree of neurologic impairment in chronic lithium toxicity correlates poorly with the serum lithium concentration, which reflects the peripheral/serum compartment. In contrast, symptoms reflect slower equilibration into the central nervous system [13,15]. Concentrations only modestly above the reference range, as in this case, can produce marked symptoms in a chronically treated patient. Symptoms may persist after the serum lithium concentration has fallen or, conversely, may resolve while the concentration remains near the upper limit of the reference range.

Third, the leukocytosis in this case is itself characteristic. Lithium is a well-recognized cause of benign leukocytosis through demargination and enhanced granulopoiesis, and a reactive leukocytosis is also common in acute illness. Here the white blood cell count fell from 17.9 to 10.5 × 10³/mm³, procalcitonin was below the limit of detection, and the only organism detected on molecular testing was human rhinovirus/enterovirus. The viral infection is unlikely to explain the focal neurologic syndrome on its own, although it may have contributed to reduced intake, and it does not warrant antibacterial therapy. Recognizing this pattern would have permitted earlier de-escalation of vancomycin and piperacillin-tazobactam.

Finally, thyroid function testing showed a suppressed TSH with an elevated free T4 in a patient carrying a diagnosis of hypothyroidism, a pattern indicating levothyroxine over-replacement. Because thyroid dysfunction in either direction can contribute to cognitive change, and because lithium itself affects thyroid function, thyroid studies should be checked in this setting and reassessed after discharge. In this patient, the neurologic syndrome tracked with the lithium concentration and volume status rather than with thyroid indices, but the finding is clinically relevant to ongoing care.

Context within the existing literature

A striking feature of this interaction is how sparsely it is documented relative to how often the two drugs are now co-prescribed. Onggo and colleagues addressed this directly in a 2025 letter to the editor reporting semaglutide-mediated lithium toxicity, published expressly to place a documented case in the peer-reviewed record at a time when the interaction circulated largely as a clinical anecdote and informal report rather than as citable evidence, and thereby to make systematic investigation possible [18]. That framing explains the apparent paradox facing any clinician who searches this topic: the scarcity of publications reflects the absence of systematic surveillance rather than a reliable estimate of how often the event occurs. Whether the interaction is common or rare therefore remains unknown, and each additional well-characterized case carries disproportionate weight without denominator data. This report is offered in that spirit.

The published literature now comprises a small number of reports. Al-Soleiti and colleagues described a series of three patients with bipolar disorder started on semaglutide, two of whom developed toxicity, and noted that theirs was among the first documented case series of this interaction [19]. Arriola Montenegro and colleagues reported lithium toxicity attributed to semaglutide in a patient with chronic kidney disease, a population in which reduced renal reserve amplifies any additional prerenal insult [20]. Burson and Leung described toxicity following a change from semaglutide to tirzepatide, indicating that the risk is not confined to a single agent and that transitions between agents constitute a distinct period of vulnerability [21]. Seth and Ramanathan reported a further case associated with tirzepatide and proposed monitoring recommendations for co-prescription [22]. To our knowledge, the present report is among fewer than ten published accounts.

Two observations from that literature deserve comment. First, the Burson and Leung case developed toxicity without vomiting, diarrhea, or a measurable change in renal function, which suggests that volume depletion, while the most readily demonstrated mechanism, may not be the only one operating; delayed gastric emptying, altered absorption kinetics, or changes in body composition and volume of distribution during rapid weight loss may contribute in patients without overt gastrointestinal losses. Second, this case provides what earlier reports largely lacked: serial renal and hematologic data across the toxic and recovered states. The fall in creatinine from 1.16 to 0.79 mg/dL, the rise in estimated glomerular filtration rate from 51 to 81 mL/min/1.73 m², and the parallel fall in serum lithium together provide laboratory support for a volume-related mechanism rather than inferring it from history alone.

Beyond individual cases, formal signal detection is essentially absent. No dedicated disproportionality analysis of GLP-1 receptor agonists co-prescribed with lithium has been published using the FDA Adverse Event Reporting System, VigiBase, or EudraVigilance, and existing GLP-1 receptor agonist pharmacovigilance work has focused on other signals such as pancreatitis, acute kidney injury, and neuropsychiatric outcomes. The most relevant systematic review of GLP-1 receptor agonist drug-drug interactions did not identify lithium as a studied agent [26]. This is a tractable and worthwhile gap: the exposure is common, the outcome is discrete and laboratory-confirmable, and the necessary data already exist in spontaneous reporting systems and claims databases.

Implications for practice

These suggestions are hypothesis-generating, based on a single case and a small amount of published literature, and require confirmation before adoption as monitoring standards. Before initiating a GLP-1 receptor agonist in a patient on lithium, document a baseline lithium concentration, basic metabolic panel, and estimated glomerular filtration rate. During dose titration, and again during any period of rapid weight loss or significant gastrointestinal intolerance, recheck the serum lithium concentration and renal function. No evidence-based monitoring interval exists for this specific scenario. Still, we propose rechecking one to two weeks after each dose increase, an interval chosen so that testing falls after gastrointestinal symptoms and volume losses typically peak following escalation but before the next scheduled increase. Patients and families should be counseled explicitly that vomiting, diarrhea, or markedly reduced oral intake are reasons to contact the prescriber rather than simply continuing therapy without medical guidance and should be taught to recognize tremor, confusion, and slurred speech as warning signs. Prescribers should watch for additive risk with concurrent thiazides, renin-angiotensin system blockers, and nonsteroidal anti-inflammatory drugs [24]. Where clinically feasible, coordination between the prescriber of the GLP-1 receptor agonist and the psychiatrist managing lithium is important. This is not a theoretical concern: prescribing data show that the growth in GLP-1 receptor agonist use among patients with mood disorders has been driven by primary care clinicians and nurse practitioners rather than psychiatrists [9,10]. Hence, the clinician starting the drug is frequently not the clinician who monitors lithium levels, and the interaction falls squarely in the gap between them. A brief note to the lithium prescriber at GLP-1 initiation, or a standing order for a lithium level and basic metabolic panel two weeks after each dose increase, would address most of this risk at negligible cost.

Limitations

This is a single case, and causality cannot be established. The timing of the last lithium dose relative to sampling was not recorded so that no lithium concentration can be confirmed as a predose trough; no lithium concentration was available from the month before presentation, and no formal measurement of lithium clearance or fractional excretion was performed. A dosing weight of 75.3 kg was recorded at presentation. Still, no pre-semaglutide or discharge weight, orthostatic vital signs, urine output, intake and output, serum osmolality, or urine sodium were available, so the degree of volume depletion is inferred from laboratory surrogates. Data were unavailable on total volume and timing of intravenous fluids, semaglutide titration history, whether semaglutide was continued after discharge, the specimen type used for the hospital day 2 respiratory panel, and the final blood and respiratory culture results. Several other factors may have contributed independently: concurrent rhinovirus/enterovirus infection may have reduced intake, biochemical levothyroxine over-replacement may have affected mentation, and iodinated contrast administered for CT angiography may have contributed to the transient decline in renal function. Chronic lithium exposure is itself associated with a progressive decline in glomerular filtration in a subset of patients [34,35], and no baseline creatinine from before the semaglutide period was available, so a contribution of pre-existing lithium-related nephropathy cannot be excluded. However, the prompt recovery with volume repletion argues that the dominant process was prerenal. Because lithium withdrawal and volume repletion occurred together, symptom resolution cannot be attributed to any change in semaglutide exposure and does not represent a semaglutide dechallenge. Even so, the temporal relationship, the unrevealing evaluation for alternative causes, the laboratory evidence of reversible renal impairment, and symptom resolution with lithium cessation and volume repletion make a semaglutide-associated interaction a plausible explanation.

## Conclusions

Symptomatic lithium toxicity developed one month after initiation of semaglutide in a patient whose lithium dose had been stable for years, presenting as a stroke mimic with dysarthria and expressive aphasia. Serial laboratory data showed a transient reduction in estimated glomerular filtration rate that improved with volume repletion, consistent with reduced renal lithium clearance in the setting of gastrointestinal intolerance and weight loss as a plausible mechanism. Clinicians should check a lithium concentration early in any patient on lithium presenting with acute neurologic change. Obtaining baseline lithium and renal function before starting a GLP-1 receptor agonist, monitoring during titration and rapid weight loss, and counseling patients on hydration and toxicity symptoms are reasonable precautions that warrant confirmation. Because the published literature on this interaction remains limited to a small number of cases and no formal pharmacovigilance analysis has been undertaken, continued case reporting and database-level signal detection are both warranted.
